# Genetic association meta-analysis: a new classification to assess ethnicity using the association of MCP-1 -2518 polymorphism and tuberculosis susceptibility as a model

**DOI:** 10.1186/s12863-015-0280-2

**Published:** 2015-10-30

**Authors:** Tania Vásquez-Loarte, Milana Trubnykova, Heinner Guio

**Affiliations:** Laboratorio de Biotecnología y Biología Molecular, Instituto Nacional de Salud, Avenida Defensores del, Morro 2268, Lima 9, Peru

**Keywords:** Polymorphism, CCL2, MCP-1, Tuberculosis, Ethnicity

## Abstract

**Background:**

In meta-analyses of genetic association studies, ancestry and ethnicity are not accurately investigated. Ethnicity is usually classified using conventional race/ethnic categories or continental groupings even though they could introduce bias increasing heterogeneity between and within studies; thus decreasing the external validity of the results. In this study, we performed a meta-analysis using a novel ethnic classification system to test the association between *MCP-1* -2518 polymorphism and pulmonary tuberculosis. Our new classification considers genetic distance, migration and linguistic origins, which will increase homogeneity within ethnic groups.

**Methods:**

We included thirteen studies from three continents (Asia, Africa and Latin America) and considered seven ethnic groups (West Africa, South Africa, Saharan Africa, East Asia, South Asia, Persia and Latin America).

**Results:**

The results were compared to the continental group classification. We found a significant association between *MCP-1* -2518 polymorphism and TB susceptibility only in the East Asian and Latin American groups (OR 3.47, *P =* 0.08; OR 2.73, *P =* 0.02). This association is not observed in other ethnic groups that are usually considered in the Asian group, such as India and Persia, or in the African group.

**Conclusions:**

There is an association between *MCP-1* -2518 polymorphism and TB susceptibility only in the East Asian and Latin American groups. We suggest the use of our new ethnic classification in future meta-analysis of genetic association studies when ancestry markers are not available. This new classification increases homogeneity for certain ethnic groups compared to the continental classification. We recommend considering previous data about migration, linguistics and genetic distance when classifying ethnicity in further studies.

## Background

Tuberculosis disease (TB) is a major public health problem worldwide. To create new strategies that will improve TB control, we need a better understanding of the biological, environmental, social, and ethnic factors [1]. One promising route is the study of polymorphisms involved in pulmonary TB susceptibility [2, 3]. Several human genes have been associated with TB development [4–6], including the *m*onocyte *c*hemoattractant *p*rotein *1* (*MCP-1*), also called *CCL2. MCP-1* belongs to a group of CC chemokines located in chromosome 17q11.2. MCP-1 protein interacts with chemokine C-C motif receptor 2 (CCR2) to activate and recruit monocytes, macrophages, CD4+ T cells and immature dendritic cells to the site of infection [7–9]. The presence of MCP-1 protein in an adequate concentration is important for granuloma formation and *M. tuberculosis* clearance [10, 11].

Although there are more than ten genetic polymorphisms in the *MCP-1* promoter and coding region, only the *MCP-1* -2518 A/G allele (reference sequence 1024611) is functional and affects gene expression [10]. A substitution from A to G in -2518 position of the promoter region increases the levels of MCP-1. This action decreases the concentration of IL-12p40, which recruits and activates memory/effector Th1 cells, thus impairing long-term protection to intracellular pathogens [10]. Observational studies have shown that *MCP-1* -2518 A/G polymorphism is associated with the development of pulmonary tuberculosis (pTB) and could be a potential marker for latent TB and disease severity [3, 12]. However, this association is different among countries such as Persia, India, Korea and China, which share continental groups [13, 14].

Geographical distribution by continents is the conventional way to assess ethnicity in meta-analysis of genetic association studies. However, population genetics has demonstrated that ethnic composition is related more with genetic distance, migration and linguistic origins rather than continental groups. In terms of ancestry biomarkers, continental grouping relies on markers such as Y-DNA and mtDNA haplogroups and varies within continents [15, 16]. As a consequence, conventional classification might introduce bias and increase heterogeneity between and within studies, decreasing the external validity of the results. Thus, it is questionable if the conventional classification is an appropriate proxy for ethnicity.

In order to have a better understanding of the relationship between ethnicity and the susceptibility to infectious diseases such as TB, we evaluated the association between the *MCP-1* -2518 A/G polymorphism and pTB susceptibility using a new multi-factorial ethnic classification and compared it with the conventional approach of continental groups. This new classification is based on previous research on genetic distance, migration and linguistic origins [16–19], which improves the homogeneity of ethnic groups. We believe that our new classification for ethnicity offers a more robust approach to explain susceptibility to disease, and that it can increase the internal validity of genetic studies when ancestry markers are not available.

## Methods

### Search strategy

**Fig. 1:**
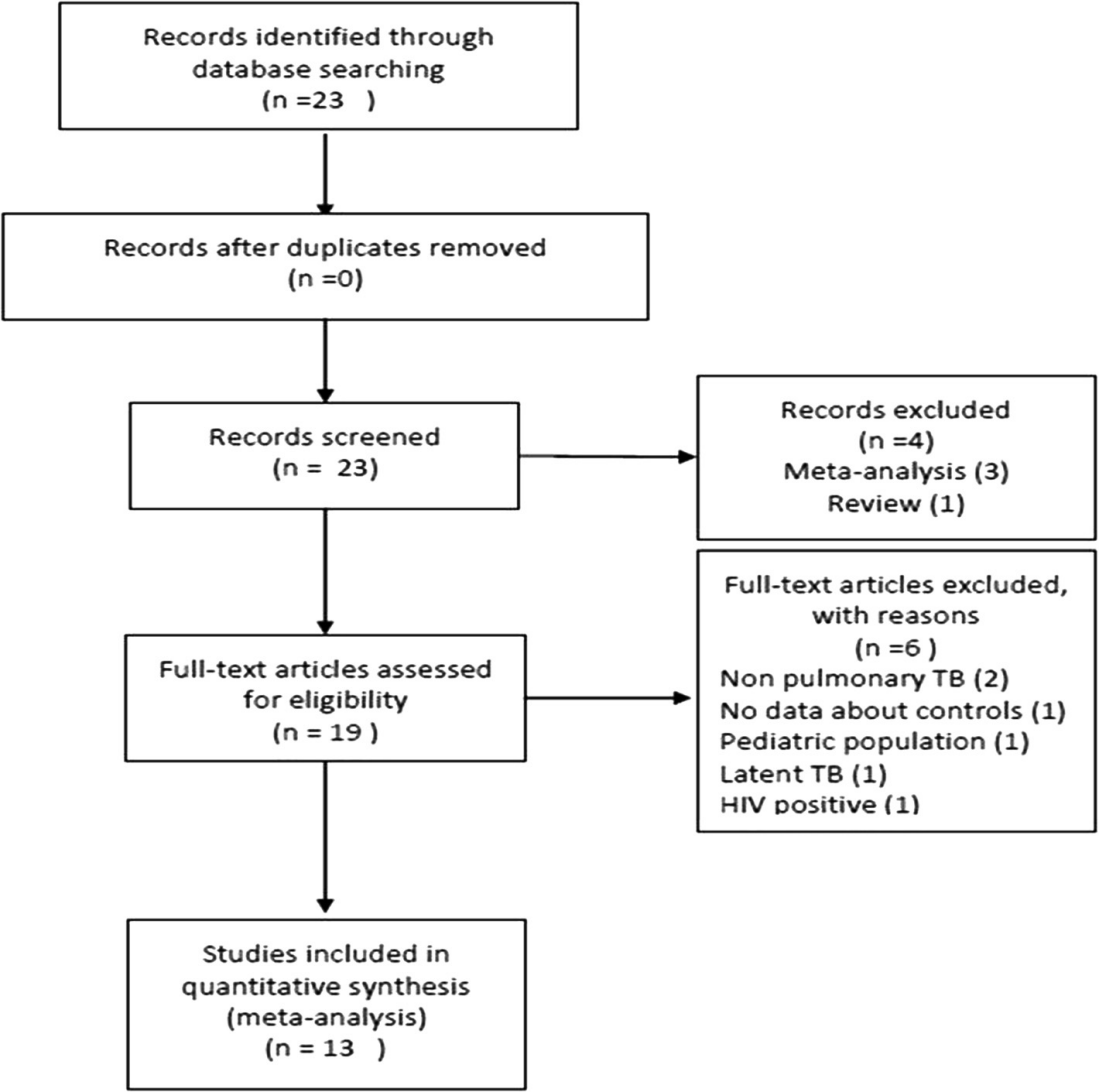
Flow chart of the selection of studies and specific reasons for exclusion from the meta-analysis. TB = tuberculosis; CCL2 = (C-C motif) ligand 2; MCP-1 = monocyte chemoattractant protein; HIV = human immunodeficiency virus

A literature search was carried out in NCBI database, Scielo and Lilacs to identify genetic association studies between MCP-1 polymorphism and pTB risk prior to December 2013. We used the following MESH terms: (("Polymorphism, Genetic"[Mesh]) AND "Chemokine CCL2"[Mesh]) AND "Tuberculosis"[Mesh]. Mesh term “MCP-1” gave the result CCL2. “Reference sequence 1024611 A/G” gave zero results. Our selection criteria included: 1) studies evaluating the association between MCP-1-2518 A/G and TB risk, 2) observational studies, 3) pulmonary TB, 4) studies performed in adults and children, 4) patients without HIV or cancer, 5) available allelic and the genotype frequencies to estimate an odds ratio (OR), 6) control groups that met Hardy Weinberg Equilibrium, and 7) articles published until December 2013. Studies that did not meet these criteria were excluded. When original articles included more than one study population, we considered each as an independent study. In case of multiple publications on the same study, we included the study with the larger sample and/or the most recently published. The data search retrieved 23 articles. Ten studies were excluded because they were reviews or meta-analyses, or corresponded to pediatric populations, spinal TB, latent TB, HIV positive individuals or data from controls was inaccessible. At the end, 13 studies (7651 cases and 8056 controls) [3, 10, 20–30] (Fig. 1) were considered.

### Data collection

All articles were separately extracted, reviewed and collated by two independent reviewers who checked for any discordance and reached a consensus in all items. Authors were contacted by email when we needed more information about an article. The following information was extracted for each study: author, year of publication, country of origin, ethnicity, sample size, type of study population, TB definition, allele and the genotype frequencies in cases, controls and methods. The information was systematically reviewed using STROBE and STREGA parameters [31, 32].

### Ethnic classification

We proposed a new ethnic classification based on previous information about genetic distance, migration and linguistic origins [16, 17, 33–35] and compared it to the conventional classification. The new ethnic classification considered previous findings about genetic distance [17]. For this purpose, data such as country of origin was extracted from each study. Finally our new ethnic classification included: Middle East Asia (Persia), East Asia (Korea and China), South Asia (India), Saharan region (Morocco and Tunisia), South Africa (South Africa), West Africa (Guinea-Bissau, Gambia, and Ghana) and Latin America (Peru and Mexico). The conventional classification includes three groups: Africa (37 %), Asia (43.8 %) and Latin America (18.8 %). The characteristics of each study are listed in Table 1. We hypothesized that the new classification creates ethnic groups that have more homogeneity than the groups obtained by the conventional group classification.

**Table 1:** Characteristics of studies included in the meta-analysis

Author, year, reference	Country	Male cases (%)	Age, mean (SD)	Diagnosis of cases	Control source and characteristics	Methods
Africa						
Ben-Selma et al., [23]	Tunisia	75	44(-)/-	Clinical and radiological pTB, BCG+	Healthy individuals, same community and ethnicity, BCG+	RFLP
Arji et al., [24]	Morocco	56	30(16)/38(17)	Clinical and radiological pTB, AFB+, HIV-, HBV-, HCV-	Healthy blood donors	RFLP
Möller et al., [22]	South Africa	-	-	Clinical and radiological pTB, AFB+, HIV-	Healthy individuals, same community, HIV-	SNPlex genotyping system
Thye et al., [20]	Ghana	-	-	Clinical and radiological pTB, AFB+, HIV-	Healthy individuals, TST-	Light type-based genotype
Velez et al., [21]	Guinea- Bissau	60	37(14)/36(12)	Clinical Pulmonary pTB, AFB+, HIV-	Healthy individuals, same community	Real-time PCR
Velez et al., [21]	Gambia	69	33(14)/ 29(13)	Clinical Pulmonary pTB, AFB+, HIV-	Neighbors, spouses	Real-time PCR
Asia						
Flores-Villanueva et al., [10]	Korea	67	38(-)/34(-)	Clinical and radiological pTB, AFB+, culture+, HIV-	Healthy blood donors	RFLP
Chu et al., [27]	Hong Kong	66	48(18)/31(9)	Clinical pTB, AFB+, HIV-	Healthy blood donors	RFLP
Xu et al., [29]	China	51	45(14)/42(13)	Clinical and radiological pTB, AFB+, in treatment	Healthy children	SSP-PCR
Yang et al., [28]	China	66	-	Clinical and radiological pTB, AFB+, in treatment	Surgery and Gynecology patients, no prior TB	RFLP
Naderi et al., [30]	Persia	22	50(21)/51(13)	Patients with confirmed pTB	Healthy individuals	Tetra-ARMS PCR
Mishra et al., [26]	India	69	37(7)/38(6)	AFB+ or patients under treatment	Healthy individuals, same ethnicity, AFB-	RFLP
Alagarasu et al., [25]	India	66	34(10)/31(9)	Clinical and radiological pTB, AFB+, HIV-	Healthy individuals	RFLP
Latin America						
Flores-Villanueva et al., [10]	Mexico	68	37(7)/36(7)	Clinical and radiological pTB, AFB+, culture+	Healthy neighbors, 334 TST+, 176 TST-	RFLP
Ganachari et al., [3]	Mexico	65	36(6)/37(3)	BCG+, clinical and radiological pTB, AFB+, HIV-	Healthy neighbors, TST+, HIV-	Tetra-ARMS
Ganachari et al., [3]	Peru	58	30(10)/34(9)	Clinical and radiological pTB, AFB+	Healthy individuals	Tetra-ARMS

pTB = pulmonary TB, AFB = acid fast bacilli, BCG, = Bacillus Calmette-Guérin vaccine, HIV = human immune deficiency virus, HBV = Hepatitis B virus, HCV = Hepatitis C virus, TST = tuberculosis skin test, RFLP = restriction fragment length polymorphism, Tetra-ARMS = amplification refractory mutation system-PCR, PCR = polymerase chain reaction

### Statistical analysis

For each study, the Hardy Weinberg Equilibrium (HWE) was calculated for the controls using *X*^2^ statistic. Genotypes deviated from HWE if two-sided p values were <0.05. Begg funnel plot and Egger’s test indicated publication bias if p value was <0.05. Sensitivity analysis was performed by removing one study at a time to assess the stability of the meta-analysis results.

To prove our hypothesis, we assessed heterogeneity and the magnitude of association for each ethnic group. We assessed heterogeneity by using the *χ*^*2*^ based Q test and *I*^*2*^ statistic. *P* values less than 0.01 were considered significant for heterogeneity. To assess the magnitude of association (pooled OR), in the presence of homogeneity, we used a fixed effects model (inverse variance weighted). Otherwise, we used a random effects model (DerSimonian and Laird, D + L). Pooled OR for the association between *MCP-1* - 2518 A/G polymorphism and pTB risk was determined in three steps. First, we did an allelic comparison (G vs. A) to determine the pooled OR in the overall data and by ethnic subgroups. Second, using our new ethnic classification, we analyzed four genotype models: a) recessive (GG vs. AG + AA), b) homogenous co-dominant (GG vs. AA), c) heterogeneous co-dominant GA vs. AA) and c) dominant (GG + GA vs. AA). Third, we compared these results to those obtained from the analysis using the conventional classification. Odds ratio estimates were considered significant if *P* was <0.05, and were expressed using a 95 % confidence interval (CI). When analyzing by ethnicity, we used the groups that had ≥1 degree of freedom. For our analysis, the wild type allele was A, and the risk allele was G. We did not adjust our model for environmental effects. The statistical analysis was performed using STATA 11.0. (STATA Corp, College Station, TX, USA).

## Results

**Fig. 2:**
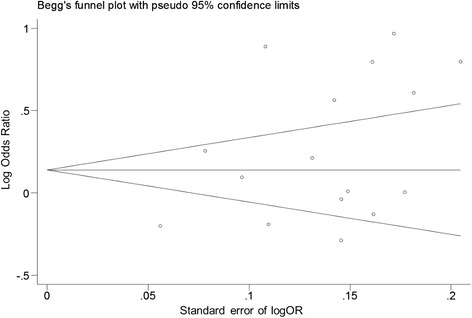
Begg’s funnel plot analysis, which detects publication bias for G allele comparison. We did not find any publication bias in the entire group analysis (t = 1.76, *P* = 0.1). OR = Odds Ratio

The conventional ethnic analysis found heterogeneity in the three continental study groups for both allelic and genotype analysis. Using our new classification, we found homogeneity for South Asia (India) and West Africa, which were further analyzed with a fixed effects model. We could not improve homogeneity within the rest of the ethnic groups and used a random effects model for their analysis. According to the Begg’s funnel plot and Egger’s test, we did not find any bias in the analysis of the entire group (t = 1.76, *P =* 0.1; Fig. 2). Sensitivity analysis did not find any prominent effect of each individual study when estimating the pooled OR. Characteristics of each study, allele and genotype distributions are shown in Tables 1 and 2 respectively.

**Table 2:** *MCP-1* allele and genotype distribution in different ethnic groups

Author	Country	Continent	Ethnic group	Cases/Controls	G allele (%) cases/controls	Cases GG	Cases AG	Cases AA	Controls GG	Controls AG	Controls AA	*P* HWE
Ben-Selma et al., [23]	Tunisia	Africa	Saharian	168/150	33.6/21.7	25	63	80	8	49	93	0.6
Arji et al., [24]	Morocco	Africa	Saharian	337/204	21.7/27.0	9	128	200	15	80	109	0.8
Möller et al., [22]	South Africa	Africa	South Africa	431/482	22.5/26.0	26	142	263	39	173	270	0.2
Thye et al., [20]	Ghana	Africa	West Africa	1964/2312	17.1/20.2	63	546	1355	92	748	1472	0.8
Velez et al., [21]	Guinea- Bissau	Africa	West Africa	314/341	25.0/21.3	17	123	174	21	103	217	0.07
Velez et al., [21]	Gambia	Africa	West Africa	236/252	24.6/24.4	18	80	138	15	93	144	0.9
Flores-Villanueva et al., [10]	Korea	Asia	East Asia	129/162	60.1/36.4	46	63	20	22	74	66	0.5
Chu et al., [27]	China	Asia	East Asia	403/461	52.1/49.8	110	200	93	113	233	115	0.8
Xu et al., [29]	China	Asia	East Asia	100/100	55.5/36.0	29	53	18	13	46	41	0.7
Yang et al., [28]	China	Asia	East Asia	167/167	68.9/50.0	84	62	21	42	83	42	0.9
Naderi et al., [30]	Persia	Asia	Middle East	142/166	29.6/29.5	17	50	75	15	68	83	0.8
Mishra et al., [26]	India	Asia	South Asia	215/294	25.1/25.9	18	72	125	20	112	162	0.9
Alagarasu et al., [25]	India	Asia	South Asia	153/203	31.4/34.2	21	54	78	29	81	93	0.1
Flores-Villanueva et al., [10]	Mexico	South America	Latin America	435/334	72.0/51.3	229	168	38	91	161	82	0.8
Ganachari et al., [3]	Mexico	South America	Latin America	193/243	68.1/54.9	93	77	23	70	127	46	0.4
Ganachari et al., [3]	Peru	South America	Latin America	701/796	70.0/64.4	354	273	74	327	371	98	0.6

### G allele frequencies in conventional and new ethnic classification

**Fig. 3:**
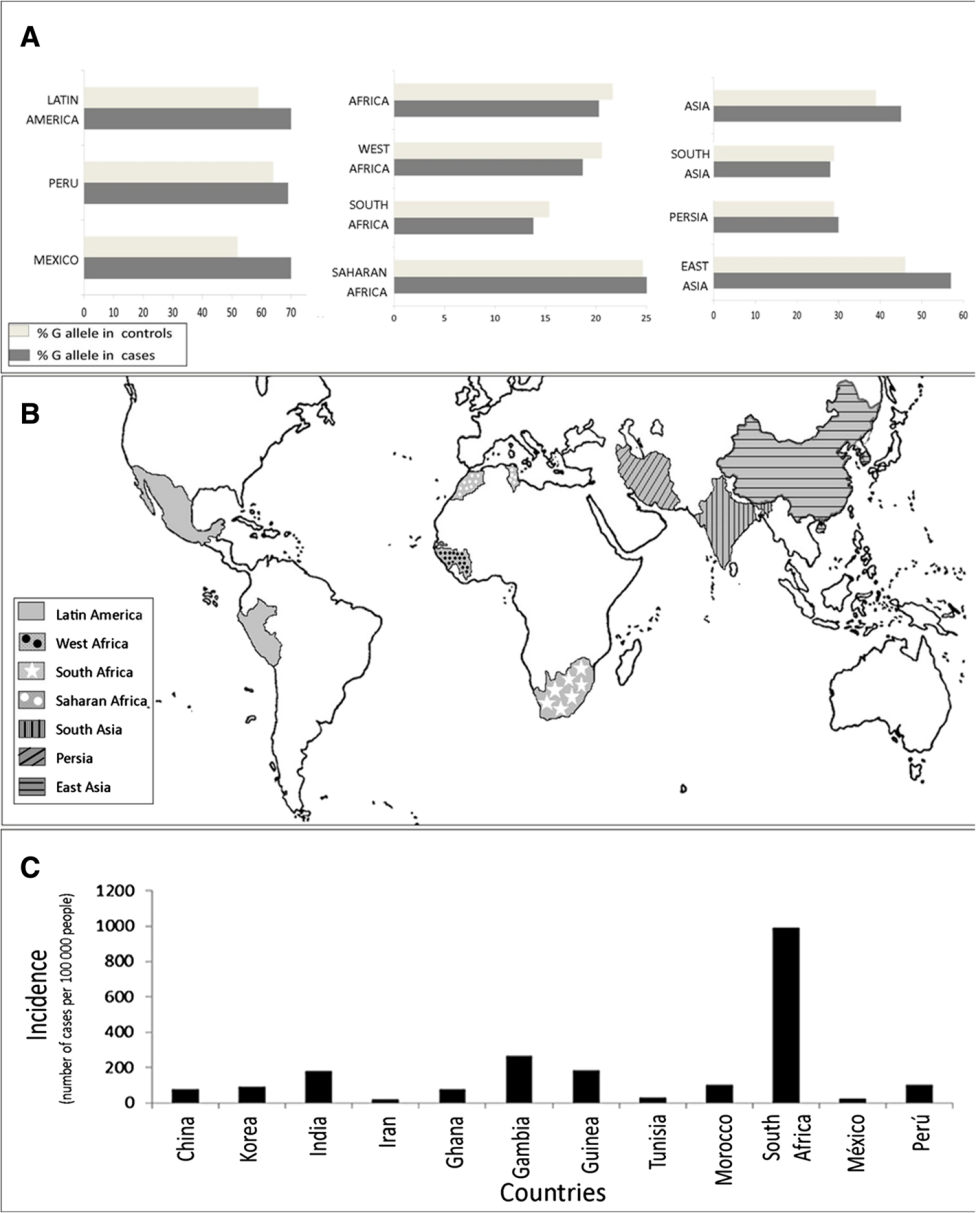
G allele *MCP-1* -2518 polymorphism distribution in study populations and incidence of pulmonary TB (2010). **a** shows the frequency of G allele among ethnic countries. G allele is more frequent in individuals with pulmonary TB from East Asian and Latin American ethnic countries (* = *P* <0.01) and there is no difference within African subgroups, Persia and South Asia. **b** shows the ethnic countries considered in our new ethnic classification. **c** The chart shows the incidence of tuberculosis in the groups studied found at http://data.worldbank.org/indicator/SH.TBS.INCD. (this incidence includes HIV cases)

**Fig. 4:**
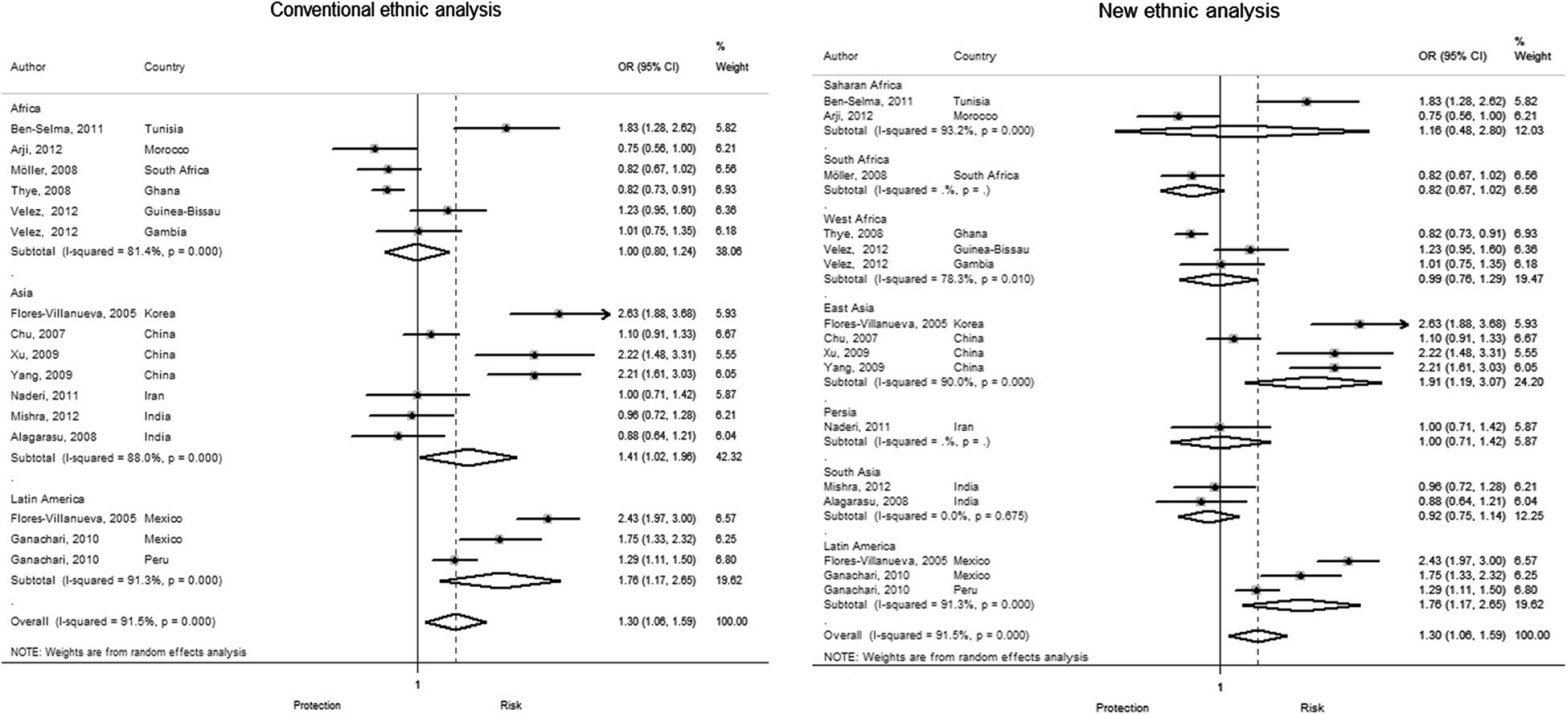
New and traditional ethnic classification to assess TB susceptibility in *MCP-1* -2518 G allele carriers. We observe that the new classification finds a significant association only for the East Asian and Latin American groups. In South Asia (India), where there is homogeneity between studies, we can rule out that the polymorphism is associated with pulmonary TB

The conventional classification showed that G allele is frequent in Asia and South America (45 % in cases vs. 39 % in controls and 70 % in cases vs. 59 % in controls, respectively) but not in Africa (22 % in cases vs. 20 % in controls). Our new ethnic classification showed that East Asia has the highest frequency of the G allele (57 % cases and 46 % controls) in the Asian group. In Latin America, this allele has a similar frequency in Mexico and Peru. In contrast, the African ethnic groups (Saharan, South and West Africa) have a low frequency of G allele in a similar proportion (Fig. 3). The pooled OR shows that presence of G allele increases the risk to develop pTB by 30 %. The conventional classification shows that this association is only significant for South America and Asia (OR 1.76, 95 % CI 1.7-2.6, *P* < 0.01; OR = 1.41,95 % CI 1.02-1.96, *P =* 0.03, respectively). Interestingly, our new ethnic classification showed that in the Asian continent, the G allele increases risk only in the East Asian ethnic group (OR 1.9, 95 % CI 1.2-3, *P* <0.01), but not for South Asian and Persia (Fig. 4). We did not find any association for any of the African groups.

### *MCP-1* -2518 A/G genotypes and pTB susceptibility

**Fig. 5:**
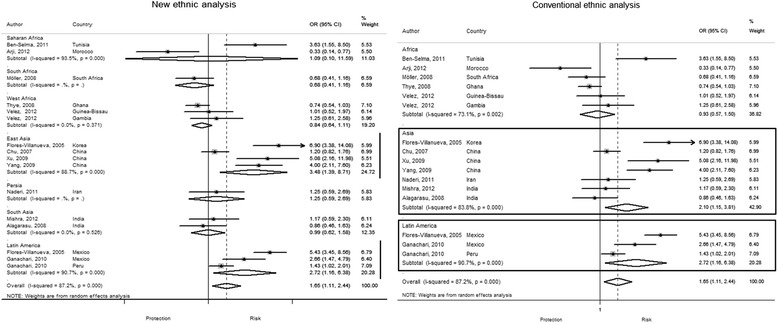
New and traditional ethnic classification to assess TB susceptibility in *MCP-1* -2518 GG genotype carriers. The homogenous co-dominant model (GG vs AA) shows that people who carry the GG genotype have 3.49 times the risk to develop pulmonary TB compared to people who have the AA genotype. The magnitude of the association in the Asian continent according to the traditional classification appears diluted because it includes South Asia and Persia, which have different ancestry and increase the heterogeneity in this continent. For Latin America, similarly to the traditional ethnic classification, we find that subjects with the GG genotype have 2.72 times the risk to develop pulmonary TB

The conventional classification showed that individuals from South America and Asia that carry GG genotype have 2.7 and 2.1 times the risk to develop pTB as compared to the ones with AA genotype (OR 2.72, 95 % CI 1.6–6.3, *P =* 0.02 and OR 2.09, 95 % CI 1.1–3.8, *P =* 0.01, respectively). The recessive model also showed increased susceptibility in both continents but to a lesser extent (OR 2.12, 95 % CI 1.5–5.4, *P* <0.01 in Asia and OR 1.76, 95 % CI 1.1–2.6, *P* < 0.01 in South America). Our new ethnic classification showed similar results for Latin America but not for Asian ethnic groups. Only the East Asian group that had the *MCP-1* -2518 polymorphism in a homozygote co-dominant and recessive model had an increased risk to develop pTB (OR 3.47, 95 % CI 1.4–8.7, *P* <0.01 and OR 2.34, 95 % CI 1.3-4.3, *P* <0.01, respectively). The new classification did not find any association for the Persian and South Asian groups. Neither the new nor the continental group classifications found any association in Africa or any of its ethnic groups (Figs. 4, 5).

### Ethnic groups and heterogeneity

We found heterogeneity within the ethnic groups from the conventional classification (*I*^*2*^ 73.1 %, *P* <0.01, for Africa; *I*^*2*^ 83.8 %, *P* <0.01, for Asia; *I*^*2*^ 90.7 %, *P* <0.01, for South America). The new ethnic classification showed homogeneity for West Africa and South Asia (*I*^*2*^ 0 %, *P* = 0.3; I^2^ 0 %, *P* = 0.5, respectively). We found heterogeneity within Arabia, East Asia and Latin America (*I*^*2*^ 93.5 %, *P* <0.01; *I*^*2*^ 88.7 %, *P* <0.01; *I*^*2*^ 91.6 %, *P* <0.01, respectively). We could not obtain results for South Africa and Persia, since there was only one study in each group.

## Discussion

We found an association between the MCP-1 -2518 polymorphism and tuberculosis susceptibility in East Asian and Latin American populations [13, 14, 36]. Previous meta-analyses try to extrapolate this association to the Asian continent. However, since there is ethnic variability within each continent, we cannot generalize this conclusion to every ethnic group. In this way, our meta-analysis groups study populations by using information about migration and linguistics to make ethnic groups more similar. Using this method, we found that an association does not apply to every country in the same continent.

Our new ethnic classification creates ethnic groups (e.g. West Africa and South Asia) with countries sharing similar characteristics. This new classification must be further evaluated with new studies related to genetic susceptibility for infectious and noninfectious diseases.

Regarding TB susceptibility, our new classification, in contrast to the conventional classification, helped to clarify that the association between MCP-1 -2518 A/G polymorphism and pTB is specific for certain populations such as East Asia and Latin America. To our knowledge, this is the first meta-analysis that uses a model of genetic susceptibility for pTB to assess if a new ethnic classification based on previous findings about genetic distance, migration and linguistic origins, improves homogeneity within each ethnic groups [13]. Thus, we propose our new classification as a good proxy when genetic markers are not available [17, 37–42].

Previous meta-analyses that use a continental group classification found an association between the *MCP-1*- 2518 A/G polymorphism and pTB, which is significant for Asia and South America [13, 14]. However, these ethnic groups include countries that are different in terms of ancestry and therefore genetic susceptibility. Our new classification helps to improve homogeneity in South Asia and West Africa. However our new classification does not help us with homogeneity in East Asia and Latin America, where we found association between polymorphism *MCP-1* -2518 G allele and pTB. Failure to reach homogeneity could be explained because of gene-gene or gene-environmental interactions. It has been reported that people carrying polymorphism *MCP-1* -2518 and *MMP-1* -1607 have a higher risk to develop severe TB [12]. The high frequency of G allele observed in cases compared to controls in both East Asia and Latin America might support the hypothesis of a similar ancestry between these two groups [43].

Recent studies from Africa show that the G polymorphism is not common among this population [21, 24]. Human population started in Africa, which means it is the oldest population, and therefore it has had the opportunity to accumulate genetic changes, such as the accumulation of -2518 MCP-1 A allele in its inhabitants that conferred protection and made it possible to adapt to hazardous environmental conditions [44]. We also have to consider other factors influencing TB susceptibility such as malnourishment, socioeconomic, environmental and health factors. The homogeneity found in West Africa cannot be completely explained in our study. We did not assess homogeneity in South Africa, since we only had one study population [22].

To deal with heterogeneity in Asia, we considered three groups: East Asia, South Asia and the Middle East [37–39]. The Asian population started from an “out of Africa” migration 50,000 years ago. It originated from two main migratory routes. The first one moved towards South Asia (India), and the second one to East Asia. Later, Central Asia was populated by Eurasian descendants. This is why we grouped Chinese and Korean populations under the East Asia group, India under the South Asia, and considered Persia under Persia. Also, these study populations have social, educational and mating habits that have that are particular to each group [16, 33, 34, 45]. Our classification groups two similar study populations from India under South Asia. Thus in this setting, it is unlikely that pTB susceptibility is due to the presence of *MCP-1* -2518 G polymorphism. In contrast to South Asia, we found an association between this polymorphism and pTB in East Asia where we also found heterogeneity. Interestingly, India (South Asia) and China (East Asia) accounted together for more than 40 % of TB cases worldwide in the last decade [46]. However the implementation of TB control strategies in China has helped decrease prevalence by 50 %, mortality rates by almost 80 % and TB incidence rates by 3.4 % per year between 1990 and 2010 [47]. Thus, even though there is a better control of TB in China, genetic factors might be playing an important role in the development of this disease. In contrast, India maintains its high incidence for the last 10 years, which might be due to a lack of social and public health control rather than genetic factors. It is difficult to assess homogeneity within Persia [48, 49] because we only had one study population. In further meta-analyses about genetic susceptibility we recommend to give a special consideration to Central Asian countries since they share European ancestry and therefore different genetic markers compared to East and South Asia [33].

Latin American ethnic groups that originated from a Han Chinese migration to South America 3000 years ago share HLA markers with this population [50]. This similarity might also explain similar frequency of *MCP-1* -2518 G allele and other genetic markers between East Asians and Latin Americans. Since Mexico and Peru share migration, common history, language routes and admixture indexes [40, 51–53], we decided to maintain them in the same ethnic group as previous meta-analyses. However, we consider that for Latin America, we should consider two groups: one of Andean and another of European origin.

The limitations in our study are very common to meta-analyses about genetic association studies. We could not consider environmental and genetic factors that influence this association because this information was not found in the original articles. Thus, for research in multifactorial diseases such as TB, we strongly recommend future studies to include information about malnourishment, socioeconomic factors, BCG and TST status, which could also help to control for heterogeneity.

We obtained homogeneity within South Asia and West Africa, where we can rule out that *MCP-1* -2518 polymorphism is associated to susceptibility. However, despite tuberculosis susceptibility found in Latin America and East Asia due to *MCP-1* -2518 polymorphism, the populations within each group are still genetically different.

Genetic association studies in populations from Persia, South Africa, South Asia, East Asia and the Americas, where infectious diseases represent a public health problem, will help assess heterogeneity in order to understand the role of ethnicity in genetic susceptibility to these diseases. In the absence of an adequate classification that groups similar genetic characteristics, suitable for understanding genetic susceptibility, our new classification might be a potential proxy for ethnic classification in meta-analysis of genetic association studies when genetic markers are not available.

## Conclusions

In summary, using this novel approach, we found an association between the *MCP-1* -2518 polymorphism and pTB susceptibility, specifically in Latin American and East Asian populations not detected by using conventional classification. We encourage the use of our new ethnic classification in further genetic association studies for infectious and non-infectious diseases.

## Abbreviations

CCL2: Chemokine (C-C motif) ligand 2
CCR2: Chemokine C-C motif receptor
HIV: Human immunodeficiency virus
HWE: Hardy weinberg equilibrium
MCP-1: Monocyte chemoattractant protein 1
MMP-1: Matrix metalloproteinase-1
mtDNA: Mitochondrial DNA
NCBI: National Center for Biotechnology Information
OR: Odds ratio
pTB: Pulmonary tuberculosis
STREGA: Strengthening the reporting of genetic association studies
STROBE: Strengthening the reporting of observational studies in epidemiology
TB: Tuberculosis
